# Etiology of lactic acidosis in malaria

**DOI:** 10.1371/journal.ppat.1009122

**Published:** 2021-01-07

**Authors:** Hendrik Possemiers, Leen Vandermosten, Philippe E. Van den Steen

**Affiliations:** Laboratory of Immunoparasitology, Department of Microbiology and Immunology, Rega Institute for Medical Research, KU Leuven, University of Leuven, Belgium; Boston College, UNITED STATES

## Abstract

Lactic acidosis and hyperlactatemia are common metabolic disturbances in patients with severe malaria. Lactic acidosis causes physiological adverse effects, which can aggravate the outcome of malaria. Despite its clear association with mortality in malaria patients, the etiology of lactic acidosis is not completely understood. In this review, the possible contributors to lactic acidosis and hyperlactatemia in patients with malaria are discussed. Both increased lactate production and impaired lactate clearance may play a role in the pathogenesis of lactic acidosis. The increased lactate production is caused by several factors, including the metabolism of intraerythrocytic *Plasmodium* parasites, aerobic glycolysis by activated immune cells, and an increase in anaerobic glycolysis in hypoxic cells and tissues as a consequence of parasite sequestration and anemia. Impaired hepatic and renal lactate clearance, caused by underlying liver and kidney disease, might further aggravate hyperlactatemia. Multiple factors thus participate in the etiology of lactic acidosis in malaria, and further investigations are required to fully understand their relative contributions and the consequences of this major metabolic disturbance.

## Introduction

Malaria is a hazardous disease caused by 1 of the 5 human *Plasmodium* species, which are *Plasmodium falciparum*, *Plasmodium vivax*, *Plasmodium ovale*, *Plasmodium knowlesi*, and *Plasmodium malariae*. It affects more than 200 million people each year, with over 400,000 deaths [1]. Most of the infections are caused by *P*. *falciparum* and *P*. *vivax* with the more pathogenic *P*. *falciparum* responsible for the majority of the malaria deaths globally. Severe malaria is a heterogenous disease with several complications that are the main cause of death and that include cerebral malaria (CM), severe malarial anemia (SMA), malaria-associated acute respiratory distress syndrome (MA-ARDS), placental malaria (PM), acute kidney injury (AKI), and metabolic disturbances. CM is a severe neurological complication responsible for many malaria-related deaths globally and is characterized by sequestration of infected red blood cells (iRBCs) in the brain, local inflammation, blood–brain barrier damage, edema, and hemorrhages [2]. SMA mainly affects young children and is thought to arise from increased destruction of parasitized and non-parasitized RBCs and decreased production of RBCs [3]. MA-ARDS is a highly lethal lung complication characterized by pulmonary inflammation and breakdown of the alveolar–capillary membrane, resulting in vasogenic edema, lung hemorrhages, and severe hypoxemia [4]. In PM, sequestration of iRBCs and infiltration of immune cells occurs within the placental intervillous spaces, which may lead to abortion, still birth, premature delivery, and low birth weight [5]. AKI can occur in up to 40% of adult patients with severe malaria and is characterized by glomerulonephritis, acute tubular necrosis, and interstitial nephritis [6]. AKI pathogenesis in malaria is not completely understood; renal microcirculation blockade due to sequestration of iRBCs, and immune-mediated glomerular injury are possible mechanisms.

Besides these organ-specific complications, metabolic disturbances like hypoglycemia and hyperlactatemia also occur in patients with malaria. Hyperlactatemia and lactic acidosis are important prognostic factors in patients with severe malaria [7,8]. Lactate levels are also increased in cerebrospinal fluid in patients with CM predicting a poor outcome [9]. Although not all consequences of lactic acidosis in malaria are known, it is clear that severe acidosis dysregulates cellular metabolism. The body responds to decreased blood pH by increasing the rate and/or depth of breathing to expel more carbon dioxide. This may result in hyperventilation and respiratory distress. Furthermore, decreased cardiac contractility and peripheral vasodilation with subsequent hypotension are known consequences of severe acidosis [10–12]. Interestingly, lactate has been described as an effector and signaling molecule with immunosuppressive effects. Lactate may induce the increased production of myeloid-derived suppressor cells, trigger tolerogenic phenotypes in dendritic cells (DCs), skew monocyte/macrophage differentiation toward M2, cause local retention of pathogenic T cells and skew CD4 T cells toward a T helper 17 (Th17) phenotype, decrease cytotoxicity of natural killer (NK) cells, and activate endothelial cells [13].

**Fig 1:**
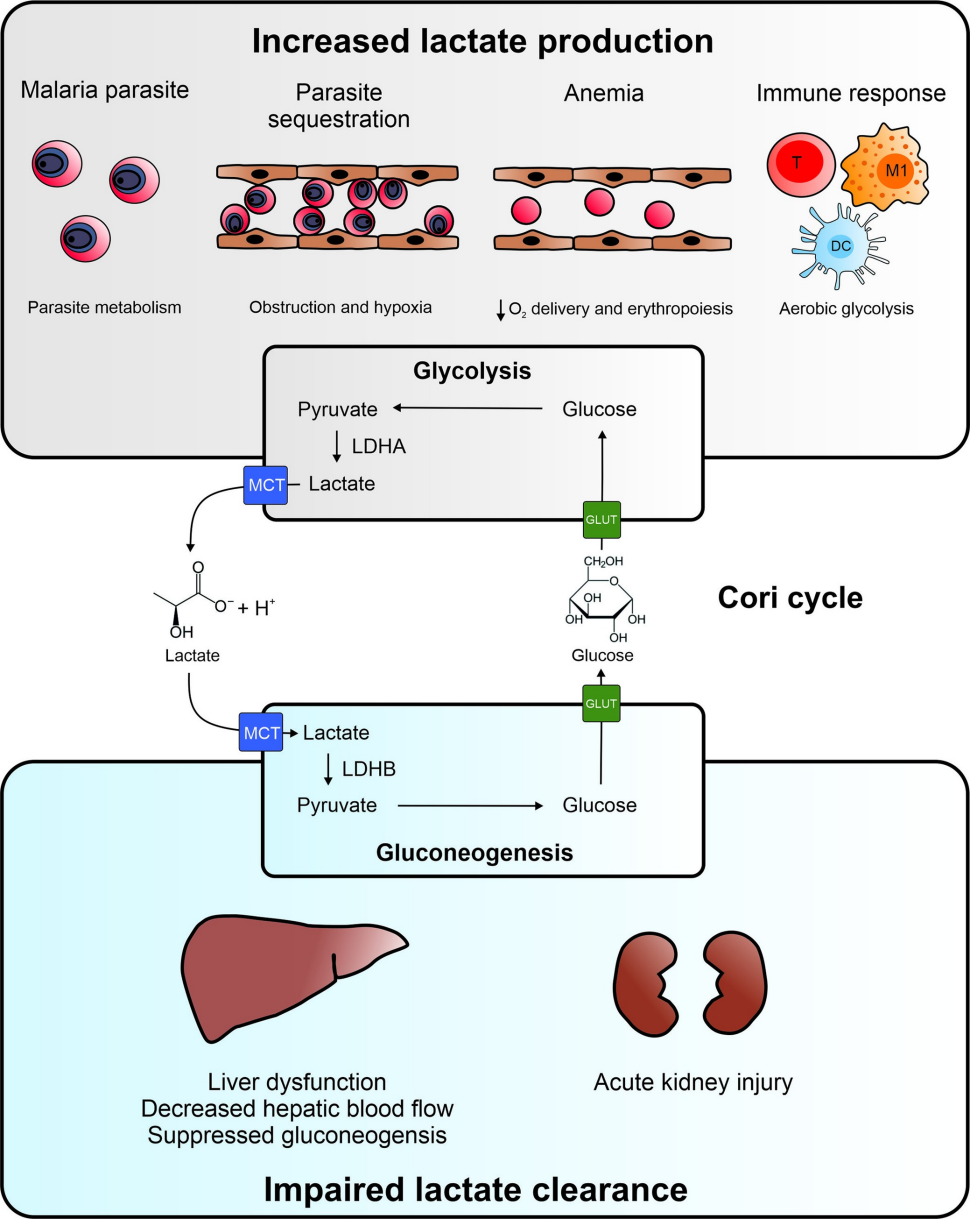
Etiology of hyperlactatemia in malaria. Both increased lactate production and impaired lactate clearance play a role in the pathogenesis of lactic acidosis. The increased lactate production might have several origins. Intraerythrocytic parasites rely on glycolysis to meet their energy requirements and produce lactate. Parasite sequestration may lead to blood vessel obstruction and induction of hypoxia in the surrounding tissues and cells, which results in increased anaerobic glycolysis. Destruction of uninfected RBCs and iRBCs causes anemia in malaria and decreases the oxygen delivery by the blood. This causes systemic hypoxia and increased anaerobic glycolysis. Malaria infection is characterized by a strong immune response with activated immune cells, which switch to aerobic glycolysis to rapidly generate ATP and metabolic intermediates for proliferation. Under all of these circumstances, glycolysis and thus the conversion of glucose into pyruvate increases. Pyruvate is further converted to lactate by LDHA, and lactate is excreted via an MCT into the circulation, which results in elevated blood lactate levels. Lactate is cleared from the circulation by uptake by gluconeogenic cells in the liver and renal cortex via MCTs. Lactate is subsequently converted to pyruvate by LDHB, and further converted to glucose via the gluconeogenic pathway. Glucose is excreted in the circulation via GLUT transporters and can be used again in the cells and tissues with increased glycolysis. This internal recycling of lactate produced by glycolytic cells and its conversion back to glucose by gluconeogenesis in the liver and kidney is known as the Cori cycle. In malaria, lactate clearance might be decreased due to liver dysfunction, decreased hepatic blood flow, and suppressed gluconeogenesis. In addition, AKI in malaria is associated with necrosis of gluconeogenic cells in the renal cortex and decreased urine excretion, which can both contribute to impaired lactate clearance. Therefore, both liver and kidney pathology can contribute to hyperlactatemia and lactic acidosis in malaria. AKI, acute kidney injury; DC, dendritic cell; GLUT, glucose transporter; iRBC, infected red blood cell; LDH, lactate dehydrogenase; M1, M1 macrophage; MCT, monocarboxylic acid transporter; RBC, red blood cell; T, T cell.

The etiology of lactic acidosis in severe malaria is incompletely understood, despite its severity and strong association with mortality. In this review, we discuss possible causes for hyperlactatemia and lactic acidosis in malaria as done in previous reviews, and we will further supplement this with the most recent findings and new hypotheses [14,15]. We thereby focus on the one hand on the increased lactate production by the parasite and the host as a consequence of parasite sequestration, anemia, and immune responses and on the other hand on the impaired hepatic and renal clearance of lactate (Fig 1).

Lactic acidosis is also common in severe non-malarial sepsis and septic shock, in which it is also associated with poor prognosis [16]. Similarities but also clear differences exist in the etiology of lactic acidosis in both conditions. Therefore, we will briefly compare the multiple causes of lactic acidosis in malaria versus non-malarial sepsis throughout the review and in Table 1.

**Table 1:** Comparison of causes of lactic acidosis in malaria versus sepsis.

Cause of lactic acidosis	Malaria	Sepsis
**Increased lactate production**		
Lactate production by the pathogen	Significant amounts, related to high parasite biomass	Negligible
Blood vessel obstruction leading to local anaerobic glycolysis	Caused by massive parasite sequestration, which is abundant in severe *falciparum* malaria	May be caused by microthrombi or by leukocytes
Microvascular dysfunction leading to disturbed blood flow	Caused by host inflammatory mediators, impaired nitric oxide bioavailability, sequestering parasites, and products released by the parasite	Caused by damage- and pathogen-associated molecular patterns, oxidative stress, and altered nitric oxide production
Anemia, leading systemically to lower oxygen delivery	Mainly caused by the destruction of RBCs by the parasite and by shortened life span of RBCs (splenic clearance of uninfected RBCs) Hemoglobin levels of <5 g/dl in SMA	Anemia of inflammation, hemodilution or bleeding Moderate anemia with hemoglobin levels rarely under 8 g/dl
Hypovolemia and hypotension	Limited contribution	A main contributor of circulatory dysfunction and subsequent hypoxia
Immune response, with aerobic glycolysis by leukocytes	Massive Th1/M1 immune response	Only in the early inflammatory phase, not in the subsequent immune suppression phase
(Nor)adrenaline stimulation of β2 adrenergic receptors, resulting in higher glycolytic flux	Usually not increased in malaria	Increased and often used as therapy
Mitochondrial dysfunction resulting in elevated anaerobic metabolism	Poorly investigated	Widespread, associated with sepsis severity and poor outcome
**Impaired lactate clearance**		
Liver dysfunction leading to decreased lactate clearance	At least in a subset of patients	At least in a subset of patients
AKI leading to decreased lactate clearance	Common in adult severe malaria patients (up to 40%)	Common (present in 1/3 of the patients)

AKI, acute kidney injury; M1, M1 macrophage; RBC, red blood cell; SMA, severe malarial anemia; Th1, T helper 1.

### The Cori cycle and lactic acidosis

Lactic acid and its anion lactate are mainly formed by the glycolysis pathway. Because the pKa of lactate is 3.8, the anion lactate is the predominant form in the human body [17]. During glycolysis, glucose is converted into 2 molecules of pyruvate, generating 2 molecules of ATP from ADP and 2 molecules of NADH from NAD^+^. In the last step, pyruvate shuttles into the tricarboxylic acid (TCA) cycle, is used as a substrate for amino acid synthesis or is converted to lactate by lactate dehydrogenase (LDH). The conversion of pyruvate to lactate provides NAD^+^, which is needed to assist glycolysis. The LDH enzyme is a tetramer comprised of LDHA and/or LDHB subunits. This results in 5 isoforms of LDH in the human body, which are expressed in different organs. The LDHA subunit has a higher affinity for pyruvate and preferentially converts pyruvate into lactate, compared to the LDHB subunit which catalyzes the opposite reaction [18]. Glycolytic tissues and cells like skeletal muscles, RBCs, brain, and renal medulla contain LDHA-rich LDH and produce more lactate. Monocarboxylic acid transporters (MCTs), which transport lactate and an associated hydrogen ion, are important for the cellular influx and efflux of lactate. These MCTs shuttle the lactate from lactate-producing cells and organs via the circulation to lactate-consuming cells and organs [19]. Lactate is converted back to pyruvate in liver and renal cortex, which express LDHB-rich LDH. Pyruvate can be metabolized in the TCA cycle via acetyl-CoA, but the majority of the lactate-derived pyruvate is reconverted into glucose via the gluconeogenesis pathway [17]. This internal recycling of lactate produced by glycolytic cells and tissues and removal via gluconeogenesis by the liver and kidney is known as the Cori cycle. In addition to the classical Cori cycle, it is important to mention that besides glucose, other molecules may be converted to pyruvate and further to lactate, including specific amino acids such as alanine and glutamine. Furthermore, lactate may also serve as a fuel for oxidative metabolism in several tissues and organs [20,21].

**Fig 2:**
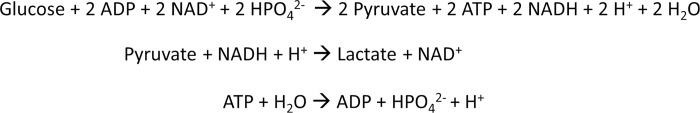
Glycolysis and LDH net reaction and acid production. During glycolysis, for 1 molecule of glucose, 2 ATP and 2 NADH are synthesized, and 2 protons are produced. In the conversion of pyruvate to lactate by LDH, NADH is converted back to NAD^+^, and an equivalent amount of protons are consumed. Upon ATP hydrolysis (e.g., for energy utilization in other reactions), protons are released, resulting in a net acidification. LDH, lactate dehydrogenase.

In the human body, 20 mmol of lactate per kilogram of body weight is produced daily. Normal blood lactate concentrations range from 0.5 to 1.5 mM and reflect the balance between the amount of lactate released into and cleared from the blood. However, when there is an increase in lactate production, a decrease in lactate removal or both, hyperlactatemia arises. Hyperlactatemia is a persistent increase of blood lactate concentration in the resting state (>2 mM) and is associated with an increase in hydrogen ions (H^+^) and with the onset of lactic acidosis (Fig 2). Lactic acidosis is mostly defined as a blood lactate concentration higher than 5 mM in combination with a blood pH lower than 7.35 [22]. Besides lactate, other organic acids lower the pH of the blood in malaria patients and contribute to acidosis [23,24].

Relevant and recent literature was collected from PubMed using search terms including lactate, malaria, hyperlactatemia, lactic acidosis, sepsis, Cori cycle, sequestration, anemia, hypovolemia, leukocyte activation, liver disease, lactate clearance, and acute kidney injury.

## Increased production of lactate

### Parasitic synthesis of lactate

In malaria, the parasite biomass may become particularly high. This comprises circulating parasites, with sometimes parasitemias higher than 10% and sequestered parasites, which may constitute an even higher parasite biomass as sequestration may occur massively in a variety of organs [25]. *Plasmodium* parasites largely depend on the host’s supply of glucose and amino acids [26]. Although *Plasmodium* parasites have mitochondria and all the enzymes of the TCA cycle are encoded in the intraerythrocytic parasites, the mitochondrial electron transport chain is not capable to perform oxidative phosphorylation, and the parasites largely rely on anaerobic glycolysis to generate ATP to meet their energy requirements [27]. iRBCs produce up to 100 times more lactate than uninfected RBCs, with 1 iRBC producing 524 fmol of lactate daily [28,29]. To prevent a cytotoxic buildup of lactate in the parasite cytosol and to maintain the anaerobic glycolysis, the parasite excretes lactate into the blood via a *Plasmodium*-specific lactate/H^+^ symporter in the parasite membrane, low-selectivity channels in the parasitophorous vacuolar membrane, host MCTs, and new anion-selective channels induced by the parasite in the erythrocytic membrane [30–32].

*Plasmodium* parasites produce both L- and D-lactate, with approximately 6% to 7% of the D-isomer [29] in contrast to the human body, which mainly produces L-lactate. It has been suggested that this might be used to determine the relative contribution of the parasite to the overall production of lactate [14,15]. Still, other sources of D-lactate exist: the methylglyoxal pathway, diet, and microbiome. Furthermore, the kinetics of D-lactate conversion and clearance might differ, since the classical human lactate transporters (e.g., MCT-1) and human LDH (LDHA or LDHB) are specific for the L-isoform, and the D-isoform can be metabolized by the enzyme LDHD [33–35]. Therefore, it remains difficult to delineate the relative contribution of the parasite to the total lactate production based on the abundancy of the different isoforms of lactate. A quantitative calculation suggests that at high parasite burden, the contribution of the parasite might be significant (S1 Appendix).

In a study with Gambian children infected with *P*. *falciparum*, a positive correlation was found between blood lactate concentrations and total and circulating parasite biomass, but not with the sequestered biomass only [36]. Thereby, the total parasite biomass was calculated based on the *Plasmodium falciparum* histidine rich protein 2 (*Pf*HRP2) concentrations in the plasma. The estimated number of sequestered parasites was calculated as the difference between the estimated total parasite biomass and the total circulating parasite biomass, the latter of which was calculated from the observed peripheral blood parasitemia [36]. This study therefore suggests that the lactate produced by the parasite itself may significantly contribute to lactic acidosis. A positive correlation between total parasite biomass and blood lactate concentration has been observed in several studies [37,38]. This further supports that parasites play an important role in the increased production of lactate, either by producing lactate itself, or by inducing lactate production in the tissues, which is discussed in the following sections.

### Parasite sequestration

Parasite sequestration is the cytoadherence of iRBCs to a variety of receptors on endothelial cells, mainly in capillaries and postcapillary venules. *P*. *falciparum* is the only species of the 5 human-infecting *Plasmodia* with a clear capability to sequester massively. Sequestration is mediated by the *Plasmodium falciparum* erythrocyte membrane protein 1 (*Pf*EMP1) adhesins and enables the parasite to escape clearance by the spleen. This results in the localized release of inflammatory molecules, but is also a unique cause of microvascular obstruction and subsequent hypoxia in the surrounding cells and tissues [39]. Local hypoxia provokes an increase in anaerobic glycolysis and thus lactate production. This has often been hypothesized to be the main cause for lactic acidosis and is specific for *P*. *falciparum* malaria, whereas bacteria or fungi in the blood during non-malarial sepsis do not specifically attach to endothelial cells [7,40–42]. While it is reasonable to assume that massive parasite sequestration induces hypoxia by obstructing blood vessels, this has not yet been formally proven, as infarction of tissue is rarely observed in malaria patients, and not all vessels in a tissue may be obstructed [43]. However, in malaria patients infected with *P*. *vivax*, a *Plasmodium* species which is unable to sequester, hyperlactatemia occurs less frequently than in patients with *P*. *falciparum* malaria [44].

Furthermore, several studies have reported an association between the sequestered parasite biomass and lactate levels. Dondorp and colleagues observed a positive correlation between the sequestered parasite biomass and the plasma lactate concentration in patients with *P*. *falciparum* malaria [37]. No association was found between the circulatory parasitemia and the plasma lactate concentration. In a study including patients with CM, sequestered parasite biomass was higher for patients with lactic acidosis compared to patients without lactic acidosis [38]. In addition, lactic acidosis in CM patients was correlated with an increased parasite biomass and decreased platelet count, which are both factors associated with parasite sequestration [38]. Hanson and colleagues visualized and quantified microvascular sequestration in the rectal mucosa of patients with severe *P*. *falciparum* malaria using orthogonal polarized spectroscopy [45]. The amount of sequestration in the rectal blood vessels correlated positively with plasma lactate concentrations. Furthermore, dual RNA sequencing of the blood of patients infected with *P*. *falciparum* found an association between lactate levels and the expression of several parasite genes important for cytoadherence [46]. These studies altogether show that sequestering parasites are associated with increased lactate levels and support the hypothesis that sequestering parasites are an important contributor to lactic acidosis, presumably through the induction of anaerobic glycolysis in the tissues. Parasite sequestration is also essential to enable a high parasite biomass in the human body, and it is therefore difficult to disentangle the parasite metabolism or the sequestration-induced hypoxic host metabolism as cause of hyperlactatemia.

In 1 study, no correlation was observed between parasite sequestered biomass and blood lactate levels in children infected with *P*. *falciparum* [36]. The authors suggested that sequestration-independent factors could play a role in hyperlactatemia, and this could also be related to the specific complications in the studied patient population.

### Anemia

In malaria, both the destruction of uninfected RBCs and iRBCs after schizont rupture play an important role in the development of anemia [47]. In anemic patients, the capacity of oxygen delivery to the tissues is reduced and in severe cases (hemoglobin <5 g/dl), this might eventually lead to tissue ischemia and hypoxia. In contrast to sequestration-induced hypoxia, which is restricted to sites of sequestration, the hypoxia in anemic patients is systemic. This may lead to an increase in systemic anaerobic glycolysis, higher lactate production, and lactic acidosis. Several studies reported an association between lactate levels and anemia in patients with malaria [40,48–50]. In these studies, the association between lactate levels and anemia was not always corrected for parasite biomass [48,50]. However, parasite biomass is typically considerably higher in patients with CM than in patients with SMA, which suggests that this correction may be less important in SMA [51]. Furthermore, Brand and colleagues observed that in patients with SMA, lactic acidosis was associated with decreased hemoglobin levels, whereas in CM patients, lactic acidosis correlated with parasite sequestration [38]. In patients with both CM and SMA, lactic acidosis was more related to hemoglobin levels than to parasite biomass, suggesting that anemia contributed to a greater extent to lactic acidosis in these patients [38].

Development of anemia normally leads to the induction of erythropoiesis in the bone marrow and release of newly formed reticulocytes in the circulation. The metabolism of erythroid progenitors, which display a high proliferation rate, depends on aerobic glycolysis leading to high lactate production under aerobic conditions, called the Warburg effect [52]. Erythropoiesis induced in anemic malaria patients may therefore produce significant amounts of lactate. However, an impaired erythropoiesis has been frequently described in anemic patients with malaria and is thought to be related with the suppressive effects of proinflammatory cytokines and nitric oxide [47]. Therefore, erythropoiesis might also be a significant contributor to lactic acidosis, but only in anemic patients without erythropoietic suppression. This needs to be further investigated.

Although rarely as severe as in SMA, anemia is also a common feature in non-malarial septic patients, and this might thus contribute to lactic acidosis to some extent. In non-malarial sepsis, anemia may be caused by iatrogenic blood loss, depression of serum iron levels and erythropoietin production, intravenous fluid administration, disseminated intravascular coagulation (DIC), and a decreased life span of erythrocytes [53–58]. These causes may also contribute to the anemia in malaria patients.

### Circulatory failure

Hypovolemia, hypotension, and shock in malaria patients may result in tissue hypoperfusion and hypoxia-induced hyperlactatemia. However, Hanson and colleagues found that lactic acidosis was worse in less hypovolemic malaria patients [59]. Hypotension and shock are observed in only a minority (approximately 12%) of the patients with severe malaria [60]. It has been suggested that hypotensive shock in patients with severe malaria could indicate concomitant bacterial sepsis, which is more frequently associated with hypotension and shock [61].

### Immune responses in malaria patients

Leukocyte activation and proliferation require several metabolic processes, which are determined by the leukocyte subtype and activation stimulus [62]. In quiescent, nonproliferating cells, the TCA cycle and oxidative phosphorylation are supported, generating high levels of ATP per molecule of glucose. Despite sufficient availability of oxygen for the TCA cycle and oxidative phosphorylation, rapidly proliferating and/or inflammatory cells switch to aerobic glycolysis to quickly generate ATP. Thereby, 1 molecule of glucose gives rise to less ATP but more metabolic intermediates that support cell growth and proliferation. Overall, an activated immune reaction stresses the metabolic homeostasis by consuming large amounts of nutrients and producing high levels of lactate. Quantitatively, an activated leukocyte has a daily lactate production of 1,757 fmol, which is more than 3 times the lactate production of an iRBC (524 fmol) [29,63].

Antimalarial immunity involves both innate and adaptive immunity, comprises extensive phagocytic activity, T lymphocyte activation, and antibody production, and is regulated by the production of several cytokines and chemokines. Dendritic cells, monocytes, and macrophages sense *Plasmodium*-derived components through their pattern recognition receptors. This is essential for the clearance of malaria infection, but excessive activation of these cells also leads to immunopathology [64]. Both monocytes and DCs undergo profound metabolic reprogramming in response to hypoxia, danger signals, and cytokines [65]. In particular, a shift toward glycolysis and fatty acid synthesis is thought to be critical for macrophages to adopt a M1 proinflammatory state [66]. The glycolytic flux also increases profoundly upon activation of DCs [67]. The activation of these innate immune cells by iRBC and parasitic products may thus lead to increased lactate production in patients with malaria.

Th1 immune reactions with interleukin (IL)-12 and interferon gamma (IFN-γ) as the main driver cytokines are essential for antimalarial immunity [68]. However, the Th1 immune reaction may also be detrimental, which was observed in the pathogenesis of complications in mouse models, including experimental CM and MA-ARDS [69,70]. Th1 immune reactions and in particular the effector T cells are characterized by aerobic glycolysis resulting in high glucose consumption and lactate production [71,72].

It is currently not clear to what extent the Th1 reaction in malaria patients quantitatively contributes to lactic acidosis. However, it should be considered that in the erythrocytic stage of malaria, the activation of leukocytes is massive and systemic. IFN-γ produced during acute malaria substantially modulates hematopoiesis in the bone marrow, an organ larger than the liver comprising approximately 5% of the total body weight and producing approximately 500.10^9^ blood cells/day [73]. Inflammation induces increased lactate production in the bone marrow [74]. The spleen is the main organ involved in the development of the immune response against malaria, characterized by a remarkable splenomegaly [75]. The spleen of patients who died of severe malaria was around 3 times larger, weighing between 150 and 400 g compared to the normal spleen weight ranging from 100 to 150 g, with expansion of both white and red pulp [76,77]. Also in mouse models, massive splenomegaly with a more than 10-fold increase in splenocyte numbers may be observed, indicating that leukocyte activation and proliferation contribute to this splenomegaly [78]. Overall, the marked increase in spleen weight and the substantial activation of the bone marrow thus illustrates the magnitude of the immune response in malaria patients. Although the quantitative aspects of the lactate production by the activated immune system in malaria remain to be established, we hypothesize that it implies a massive metabolic need and may thus also contribute to metabolic disturbances including lactic acidosis (S1 Appendix). Supporting this hypothesis, dual transcriptome analysis of systemic host–pathogen interactions in severe malaria indicated that human genes related with the immune response most strongly correlated with lactate [46].

Such Th1 and/or M1 immune activation is obviously not unique to malaria and may also occur in other infections. In non-malarial sepsis, the systemic exposure of the host to pathogens initiates an excessive inflammatory response [79]. This early hyperinflammatory phase, in which activated immune cells can produce significant amounts of lactate, is followed by a simultaneous inflammatory and anti-inflammatory state. During the anti-inflammatory state, sepsis-induced immune suppression impacts both innate and adaptive immune systems. This is characterized by defects in antigen-presenting cells (APCs) and apoptosis of lymphocytes and APCs [80,81]. Interestingly, LPS-stimulated peripheral blood mononuclear cells (PBMCs) from septic patients in the hyperinflammatory phase secrete high amounts of lactate, while LPS-stimulated PBMCs of patients in the immune suppression phase were unable to produce lactate [82]. These data suggest also that in non-malarial sepsis, immune cells may contribute to lactic acidosis during the early hyperinflammatory phase but may not do so during the immune suppression phase. Further quantitative studies are required to directly validate the role of the immune response as a cause of lactic acidosis.

## Impaired lactate clearance

### Decreased lactate clearance by the liver

Gluconeogenesis in the liver occurs with alanine, lactate, and/or glycerol as precursors and is responsible for up to 70% of the lactate removal in the human body. The tight link between glucose metabolism and lactate levels is reflected by the common co-occurrence of lactic acidosis and hypoglycemia and the fact that children with metabolic acidosis and respiratory distress are most at risk of hypoglycemia [48,83].

In healthy subjects, gluconeogenesis and glycogenolysis contribute evenly to the glucose production [84]. In normo- or hyperglycemic patients with malaria, the elevated glucose demand is met by increasing gluconeogenesis resulting in a relative contribution of 90% or even 100% to the overall glucose production [84–86]. This suggests that the availability of glucose during malaria infection mainly depends on the gluconeogenesis and thus on the consumption of its main precursors, alanine, lactate, and glycerol. Interestingly, the co-occurrence of hypoglycemia and elevated lactate and alanine levels in patients with malaria suggests that the gluconeogenesis might become insufficient to compensate for the increased glucose consumption and lactate production [87].

In malaria, jaundice occurs in up to 5% of the malaria cases in endemic regions and between 10% and 60% in areas of low malaria transmission [88]. Jaundice may be caused by intravascular hemolysis, DIC, or liver dysfunction. Liver dysfunction ranging from hyperbilirubinemia with or without mild elevation in transaminases to fulminant hepatic failure has been described in patients with *P*. *falciparum* [89,90]. In most *P*. *falciparum*–infected patients, histological evidence of liver injury is minimal. Although in some patients with severe malaria, inflammatory infiltrates, hepatocyte swelling, hemozoin deposition, loss of microvilli at the sinusoidal pole, and centrizonal necrosis have been observed in liver tissue [90].

Impaired clearance of lactate by the liver may directly influence the blood lactate concentration [91]. A positive correlation between plasma lactate levels and plasma bilirubin levels in patients with *falciparum* malaria was reported, and the majority (>95%) of *P*. *falciparum–* or *P*. *vivax*–infected patients with hyperbilirubinemia had increased serum lactate concentrations [50,92]. Moreover, levels of alanine transaminase, an enzyme of which increased circulating levels indicate a diseased liver, were associated with hyperlactatemia in adult malaria patients [50]. These studies therefore support the hypothesis that liver dysfunction contributes to increased lactate levels.

Although gluconeogenic alanine use is adequate in patients with uncomplicated malaria, alanine consumption is lower if the disease is more severe, possibly explained by decreased liver perfusion or impaired hepatic clearance [93]. A decreased hepatic blood flow might similarly impair the removal of circulating lactate by the liver, supported by the inverse correlation between the blood flow in the liver and plasma lactate concentrations in patients with malaria [94]. In addition, the anaerobic tissue environment that is created by decreased oxygen delivery and increased inflammation may favor the liver itself to convert pyruvate into lactate, thereby decreasing lactate uptake from the blood [95]. Importantly, malaria infection induces elevated cytokines, which might further suppress gluconeogenesis. Tumor necrosis factor alpha (TNF-α) for instance suppresses phosphoenolpyruvate kinase (PEPCK) and IL-6 inhibits PEPCK and glucose-6-phospatase through STAT3 as seen in in vitro and in vivo models of gluconeogenesis [96–98].

The contribution of liver dysfunction to lactic acidosis in malaria is comparable as in other conditions. In non-malarial septic patients, liver dysfunction has been associated with hyperlactatemia in hemodynamically stable patients as the result of impaired lactate clearance rather than increased production [16,99]. In contrast, Revelly and colleagues found that hyperlactatemia in non-malarial sepsis was mainly related to increased lactate production [100]. These data suggest that impaired lactate clearance may contribute to lactic acidosis in certain septic patients, most likely in patients with underlying liver disease, induced liver dysfunction or, as discussed in the following section, in patients with AKI.

Altogether, these observations indicate that in a subset of malaria patients, impaired hepatic lactate clearance because of liver dysfunction, decreased blood flow, and/or suppressed gluconeogenesis may presumably play a significant role in the development of lactic acidosis.

### Impaired renal lactate clearance

Next to the liver, the kidney is the second organ for lactate clearance via gluconeogenesis and urine excretion. In healthy persons, up to 30% of the lactate is removed via gluconeogenesis in the proximal tubules of the renal cortex [22,101]. Urinary excretion of lactate is only significant with hyperlactatemia above 6 mM [102]. Children who died from malaria showed higher levels of glutamine, a precursor for the renal gluconeogenesis, than survivors, and this correlated with lactate levels, suggesting impaired gluconeogenesis in the kidneys [103].

AKI can occur in up to 40% of the adult patients with severe malaria [104]. The main histopathological finding in malaria associated AKI is acute tubular necrosis, including necrosis of the proximal tubules [104,105]. This can significantly reduce the gluconeogenic capacity of the kidney and may thus contribute to a diminished lactate clearance and lactic acidosis. Furthermore, the decreased urine production and anuria in AKI might further impede the lactate excretion and therefore contribute to severe hyperlactatemia. In patients without malaria, it was observed that AKI leads to impaired gluconeogenesis in the kidney with a switch from net renal lactate uptake to net renal lactate release in the blood [106]. Impaired renal gluconeogenesis and lactate clearance during AKI in critically ill patients without malaria were major determinants of systemic glucose and lactate levels. In studies with patients infected with *P*. *falciparum* or *P*. *vivax*, patients with AKI had increased plasma lactate levels compared to patients without AKI [104,107,108]. However, in these studies, the patients with AKI also had increased parasite load, anemia, or higher plasma bilirubin levels, indicating liver dysfunction. These factors, as discussed above, may also contribute to hyperlactatemia and confound the association between AKI and increased lactate levels.

AKI may also be an unrecognized contributor to lactic acidosis in non-malarial sepsis, because 1 in 3 patients with non-malarial sepsis develop AKI [109]. However, in malaria, AKI has been associated with sequestration of iRBCs to glomerular and interstitial capillaries, which may even more contribute to renal ischemia and predispose to acute tubular necrosis [110]. Therefore, AKI in malaria patients may have a different pathophysiology than AKI in septic patients, which could affect the lactate clearance by the kidney.

## Gaps in current knowledge, treatment options, and future perspectives

The most important gap in the current knowledge about lactic acidosis in malaria is the quantitative contribution of the various etiologies, which remains unclear despite approximative calculations (S1 Appendix). This is further complicated by the heterogeneity of malaria pathology. Quantitative approaches are needed to study this in malaria, including detailed lactate turnover studies to discriminate increased lactate production versus decreased clearance. Only 2 such studies have been published with limited numbers of patients, suggesting that increased production was decisive [111,112]. However, both studies did not discriminate between different complications and included mainly CM cases. Furthermore, part of these patients was treated with quinine, which has a considerable influence on glucose metabolism by inducing insulin. The total lactate production in malaria patients was clearly different in both studies; this may be related to adult versus pediatric patients or to experimental differences and makes further comparisons with the production by cellular sources difficult (S1 Appendix). Such studies need to be extended to larger groups of adult and pediatric patients with informative comparisons between the different complications of malaria, uncomplicated malaria, and healthy controls. This will help to delineate more clearly the relative contribution of lactate production versus lactate clearance in the various complications. Other approaches may include detailed metabolomics and/or mechanistic studies in rodent malaria models.

A better understanding of the quantitative contribution of the various etiologies in the different subsets of severe malaria is required to develop more targeted and adequate treatments. Correction of acidic blood pH with bicarbonate or other neutralizing agents is useful to avoid the detrimental effects of acidosis, but does not remove the underlying cause. Blood transfusion might be beneficial to reduce lactate in patients with SMA, because there is already evidence that anemia is an important contributor to hyperlactatemia in this subset of patients [38]. Rapid killing of the parasite with artesunate might decrease the lactate production by the parasite. Dichloroacetate (DCA), which inhibits pyruvate dehydrogenase kinase and redirects pyruvate to the TCA cycle, has been shown to decrease lactate levels in severe malaria cases, but did not decrease the overall mortality [113]. Its beneficial effects might have been mitigated by its pro-oxidative and hepatotoxic properties [114]. Mouse model studies indicate that interventions targeting glycolysis may be beneficial in CM but may also impair disease tolerance [115,116]. Furthermore, sequestration-inhibiting agents that are currently being developed may become helpful to decrease lactic acidosis by alleviating blood vessel obstruction and subsequent tissue hypoxia, especially in patients with high sequestered parasite biomass [117].

## Conclusions

Lactic acidosis is frequently observed in patients with severe malaria and is associated with a poor prognosis. Parasite sequestration is often proposed as the main contributor to lactic acidosis. However, this review supports a more elaborate view on the etiology of hyperlactatemia and lactic acidosis, including both increased production and impaired clearance of lactate. There are both similarities and differences between the etiologies of lactic acidosis in malaria and non-malarial sepsis. The quantitative contribution of the various etiologies in the different subsets of severe malaria should be further studied in the future. The better understanding of the distinct etiologies of lactic acidosis in severe malaria is required to develop more adequate treatments.

## Supporting information

S1 Appendix Considerations on the quantitative production of lactate in malaria. (DOCX)
