# Supplementary material for: Etiology of lactic acidosis in malaria
Source: PLoS Pathog. 2021 Jan 7;17(1):e1009122. doi: 10.1371/journal.ppat.1009122 (PMC7790250; doi:10.1371/journal.ppat.1009122)
Supplement: S1 Appendix — (DOCX) [file ppat.1009122.s001.docx]

S1 Appendix: Considerations on the quantitative production of lactate in malaria

Currently, it is not well defined which are the main producers of lactate during severe malaria. Here, we provide some quantitative calculations based on literature data and on values for an average adult person. These calculations provide a framework to estimate orders of magnitude, but several limitations must be taken into account, which makes it difficult to calculate the relative contributions of the various producers of lactate in a reliable manner.

**Total lactate production in basal conditions in an adult human body:**

- 1300 mmol/day (1)
- 3370 mmol/day (2)

**Total lactate production in severe malaria:**

- 25 µmol/kg/min in adults with severe malaria = 25 µmol/kg/min x 60 min x 24 h x 65 kg = 2340 mmol/day (3)
- 94 µmol/kg/min in children with severe malaria = 94 µmol/kg/min x 60 min x 24 h x 65 kg = 8798 mmol/day (4)

🡪 Limitation: lactate production in children may not be directly comparable to adults. Patients treated with quinine were excluded

**Lactate production by iRBCs:**

- 0,524.10^-12^ mol/iRBC/day (5)
- Assuming that 10% of RBCs are infected, with a blood volume of 5 l and taking into account some level of anemia with 2,5.10^9^ RBCs/ml (reference value in an adult is 5.10^9^ RBCs/ml)

🡪 total of 1250.10^9^ iRBCs

- Total lactate production = 655 mmol/day
- Limitations: total number of iRBCs highly variable between patients and between complications

**Lactate production by hypoxic tissues:**

- Unknown

**Lactate production by erythropoetic cells:**

- Basal conditions: blood volume of 5 l, 5.10^9^ RBC/ml, RBC life span 120 days

🡪 production of 208.10^9^ RBCs/day

- In malaria: life span of uninfected may be significantly shortened, but erythropoiesis may be inhibited
- Limitations: lactate production per erythropoietic cell not known, and number of erythropoetic cells in malaria patients may be highly variable

**Lactate production by leukocytes:**

- 1757.10^-12^ mol/activated leukocyte/day compared with 288.10^-12^ mol/non-activated leukocyte/day (6)
- Bone marrow in basal conditions: production of 140.10^9^ leukocytes/day (mainly neutrophils) (7)
- Spleen: 40.10^9^ leukocytes/spleen in basal conditions (8), spleen size x3 (including white pulp expansion) in malaria (9,10)

🡪 120.10^9^ leukocytes/spleen

- Assuming a total of 260.10^9^ activated or proliferating leukocytes:
- Total lactate production = 457 mmol/day
- Limitations: total number of activated or proliferating leukocytes not effectively known and may differ significantly between patients and complications, and the amount of lactate produced per activated leukocyte may differ between the leukocyte subtypes (lymphocytes, macrophages, granulocytes,…)

**References**

1. Phypers B, Pierce JMT. Lactate physiology in health and disease. Contin Educ Anaesthesia, Crit Care Pain. 2006;6(3):128–32.

2. Cowett R, Wolfe R. Glucose and lactate kinetics in the neonate. J Dev Physiol. 1991;16(6):341–7.

3. Davis TME, Binh TQ, Thu LTA, Long TTA, Johnston W, Robertson K, et al. Glucose and lactate turnover in adults with falciparum malaria: Effect of complications and antimalarial therapy. Trans R Soc Trop Med Hyg. 2002;96(4):411–7.

4. Agbenyega T, Angus BJ, Bedu-Addo G, Baffoe-Bonnie B, Guyton T, Stacpoole PW, et al. Glucose and lactate kinetics in children with severe malaria. J Clin Endocrinol Metab. 2000;85(4):1569–76.

5. Vander Jagt DL, Hunsaker LA, Campos NM, Baack BR. d-Lactate production in erythrocytes infected with Plasmodium falciparum. Mol Biochem Parasitol. 1990;42(2):277–84.

6. Haji-Michael PG, Ladrière L, Sener A, Vincent JL, Malaisse WJ. Leukocyte glycolysis and lactate output in animal sepsis and ex vivo human blood. Metabolism. 1999;48(6):779–85.

7. Fliedner TM, Graessle D, Paulsen C, Reimers K. Structure and function of bone marrow hemopoiesis: Mechanisms of response to ionizing radiation exposure. Cancer Biother Radiopharm. 2002;17(4):405–26.

8. Li ZF, Zhang S, Lv GB, Huang Y, Zhang W, Ren S, et al. Changes in count and function of splenic lymphocytes from patients with portal hypertension. World J Gastroenterol. 2008;14(15):2377–82.

9. Urban BC, Hien TT, Day NP, Phu NH, Roberts R, Pongponratn E, et al. Fatal Plasmodium falciparum malaria causes specific patterns of splenic architectural disorganization. Infect Immun. 2005;73(4):1986–94.

10. Buffet PA, Safeukui I, Deplaine G, Brousse V, Prendki V, Thellier M, et al. The pathogenesis of Plasmodium falciparum malaria in humans: Insights from splenic physiology. Blood. 2011;117(2):381–92.
